# Hybrid Polystyrene–Plasmonic Systems as High Binding Density Biosensing Platforms

**DOI:** 10.3390/ijms25168603

**Published:** 2024-08-07

**Authors:** Charles M. Darr, Juiena Hasan, Cherian Joseph Mathai, Keshab Gangopadhyay, Shubhra Gangopadhyay, Sangho Bok

**Affiliations:** 1 Department of Chemical and Biomedical Engineering, University of Missouri, Columbia, MO 65211, USA; 2 Department of Electrical and Computer Engineering, University of Denver, Denver, CO 80210, USA; 3 Department of Electrical Engineering and Computer Science, University of Missouri, Columbia, MO 65211, USA

**Keywords:** polystyrene, plasmonic gratings, COVID-19, single-molecule counting, fluorescence, biosensors

## Abstract

Sensitive, accurate, and early detection of biomarkers is essential for prompt response to medical decisions for saving lives. Some infectious diseases are deadly even in small quantities and require early detection for patients and public health. The scarcity of these biomarkers necessitates signal amplification before diagnosis. Recently, we demonstrated single-molecule-level detection of tuberculosis biomarker, lipoarabinomannan, from patient urine using silver plasmonic gratings with thin plasma-activated alumina. While powerful, biomarker binding density was limited by the surface density of plasma-activated carbonyl groups, that degraded quickly, resulting in immediate use requirement after plasma activation. Therefore, development of stable high density binding surfaces such as high binding polystyrene is essential to improving shelf-life, reducing binding protocol complexity, and expanding to a wider range of applications. However, any layers topping the plasmonic grating must be ultra-thin (<10 nm) for the plasmonic enhancement of adjacent signals. Furthermore, fabricating thin polystyrene layers over alumina is nontrivial because of poor adhesion between polystyrene and alumina. Herein, we present the development of a stable, ultra-thin polystyrene layer on the gratings, which demonstrated 63.8 times brighter fluorescence compared to commercial polystyrene wellplates. Spike protein was examined for COVID-19 demonstrating the single-molecule counting capability of the hybrid polystyrene-plasmonic gratings.

## 1. Introduction

Body fluids such as urine, saliva, and sweat often carry biomarkers that offer a noninvasive means for disease detection and identification [1,2]. However, whereas biomarkers for some pathologies are detectable at physiologic levels without difficulty, cancers and some infectious diseases are deadly even in small quantities and require early detection to prevent significant impacts to patient health [3,4]. The scarcity of these biomarkers in the early stages of disease necessitates signal amplification before precise measurements can be taken. One of the most common forms of signal amplification is the nucleic acid amplification test (NAAT), a class of diagnostic tests wherein target pathogen-specific DNA or RNA are amplified by polymerase chain reaction (PCR) [5,6,7]. However, NAAT sensors are only useful in the scenario wherein (1) genetic material can be used to detect the disease state and (2) sufficient unique genetic material exists in sufficient concentration to result in detectable signal post-amplification. On the other hand, sensors with built-in signal enhancement capabilities can amplify the signal coming from sparsely distributed molecules of any type without necessitating preliminary sample pre-concentration or amplification. One approach to enhance optical sensor signals involves leveraging the surface plasmon resonance (SPR) phenomenon occurring at the interface between light and a metal–dielectric interface [8]. SPR-based sensors excel at detecting biomolecules at incredibly low concentrations. They employ various mechanisms, such as measuring SPR coupling angle shifts upon biomolecule binding, as well as SPR-induced fluorescence and Surface Plasmon-Coupled Emission (SPCE) from fluorophore-labeled biomolecules. 

Our recent development, a grating-based SPR biosensor, can detect individual molecules of target biomarkers using an upright microscope in epifluorescence mode [9,10,11,12,13]. This technology has significantly amplified signal detection in various biological applications, including the study of Holliday Junction DNA hybrids [14], cancer cell imaging [15], and the detection of tuberculosis disease biomarker lipoarabinomannan at the single molecule level [16,17,18]. Surface modification of the alumina capping layer offers the opportunity to employ various surface binding methods, including the commonly used carbodiimide crosslinker chemistry involving 1-ethyl-3-[3-dimethylaminopropyl]carbodiimide (EDC) and *N*-hydroxysulfosuccinimide (Sulfo-NHS). However, the plasma processing technique required to create the necessary carbonyl groups for EDC coupling has the drawback of producing an unstable and highly reactive surface that degrades rapidly in ambient conditions. Therefore, it becomes crucial to promptly conduct the binding assay immediately after functionalization. Discovering a more stable binding surface with a simpler binding procedure would enhance the longevity of this biosensor, making it more valuable to a broader spectrum of users and applications. 

Polystyrene has long been favored as a versatile material for high-density cell and protein binding substrates for in vitro biological research [19] due to its well-established chemical properties, resistance to and compatibility with biological fluids, and ease of manufacturing techniques. While conventional methods for binding biomolecules on polystyrene surfaces have been effective, there has been a growing interest in enhancing protein immobilization on the polystyrene surface through surface modification techniques using functional groups. These methods encompass gamma (γ)-irradiation [20], acid treatment [21], oxygen plasma treatment [22], copolymerization [23], doping with quantum dots [24], and pre-immobilization with proteins [25]. Among these methods, γ-irradiation yields a surface with high bioactivity due to the formation of readily oxidized free radical carbons during the irradiation process. This results in a partially oxidized, primarily hydrophobic surface, which attracts hydrophilic residues of folded proteins to the surface while promoting binding through hydrophobic interactions [20]. 

**Figure 1:**
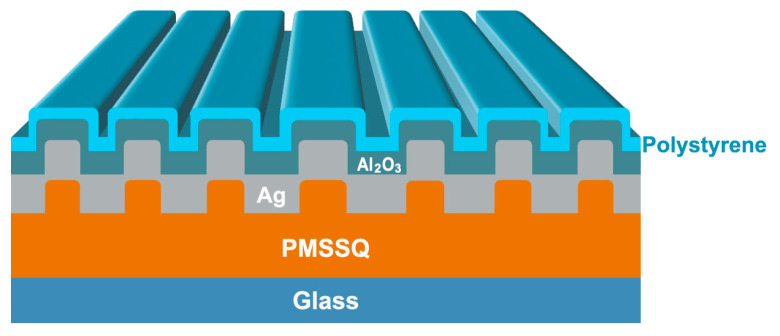
The structure of plasmonic gratings consists of Ag, Al_2_O_3_, and polystyrene on top of PMSSQ.

However, several physical factors influence and limit the thickness of a polystyrene layer on the gratings and the polystyrene–alumina interface. Electric field enhancement due to surface plasmon resonance (SPR) coupling diminishes with increasing distance from the metal–dielectric interface [9]. Consequently, the total dielectric stack above the silver layer must be minimized. The thickness of the alumina layer above the silver has been predetermined based on previous research and, so, the polystyrene film thickness should remain ideally less than 10 nm. However, this introduces an additional challenge, as films thinner than 100 nm often form isolated, discontinuous patches. This tendency is particularly pronounced when there is a mismatch in surface energy, leading to spinodal dewetting as the nascent film droplets attempt to reduce surface tension by moving away from each other and from the substrate layer [26]. Once placed in a complex aqueous sample or biofluid, unadhered surfaces would delaminate and lift off, removing the bioactive material. Functional organosilanes have been demonstrated to suppress dewetting from silica surfaces but required thermal treatment to cure the silane–polymer interface at temperatures that could destroy the sensitive silver layer [27,28]. The final structure of the plasmonic grating in this study is depicted in Figure 1. 

To overcome these challenges, the present work employs phenyl-terminated silanes to enhance adhesion between the alumina layer on top of the gratings and the bioactive polystyrene layer. This approach has allowed for the creation of a high molecular binding density through the nanostructured polystyrene thin film, stabilized by the silane-based adhesion layer, and further enhanced through surface plasmon resonance (SPR) coupling for both excitation and emission wavelengths, making it an effective chem/bio-assay platform. 

## 2. Results

### 2.1. Characterization of Thin Films

#### 2.1.1. Thickness

**Figure 2:**
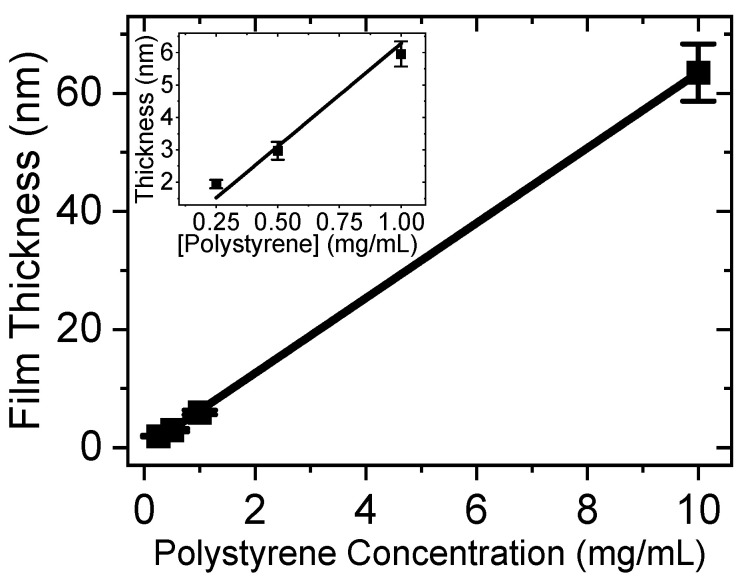
The relationship between the film thickness and the concentration (Inset: the relationship in low concentration).

The thicknesses of ultra-thin polystyrene films on silanized alumina on plain glass samples were determined by utilizing variable angle spectroscopic ellipsometry (VASE, J.A. Woollam) and were subsequently confirmed through optical profilometry (Veeco). A film thickness of approximately 63 nm was obtained when the 10 mg/mL stock solution was spun at 3000 rpm for 30 s as shown in Figure 2. Diluting the solution to 1 mg/mL resulted in a 5.9 nm (hereafter referred to as “5 nm”) thickness, while a concentration of 0.5 mg/mL produced approximately 2.9 nm. 

Notably, the film thickness was unaffected by increasing vacuum annealing time from 0 to 12 h at room temperature, indicating that the film properties were essentially set during the spin-casting process. As a result, we opted to utilize the room-temperature-annealed films for all further testing.

#### 2.1.2. Film Adhesion

**Figure 3:**
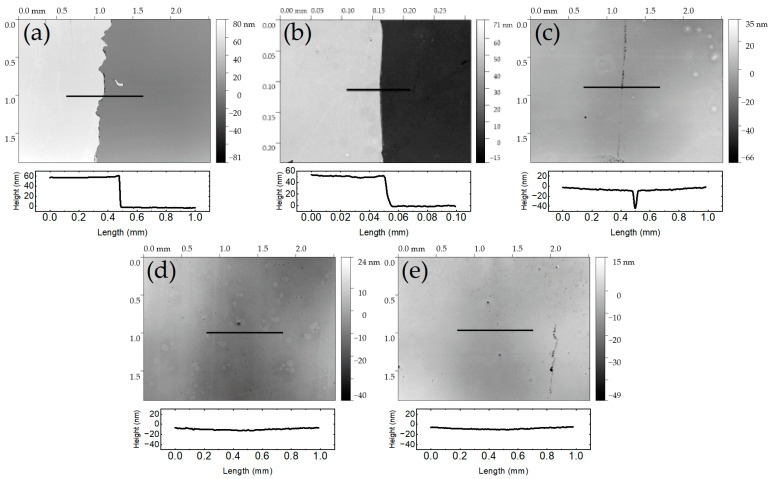
Optical profilometry after tape test of thin polystyrene films over (**a**) unsilanized alumina, (**b**) TMCS, (**c**) P Silane, (**d**) N Silane, and (**e**) G Silane.

Adhesion between the ultra-thin polystyrene layer and the alumina layer plays a crucial role in our current work. Figure 3 presents optical profilometer images and corresponding profiles for thin polystyrene films with various adhesion layer conditions subjected to a tape test. While optical profilometry is capable of nondestructively examining topographical features like thickness and roughness, the method is sensitive to the relative dielectric constants of the layers involved. Detecting height variations at the interface between materials with opposing dielectric constants, such as metal-to-dielectric materials, can be challenging, especially when dealing with dielectric films on highly reflective metal surfaces. Thus, a surrogate substrate of 10 nm alumina on plain glass was used, with polystyrene deposited using the same parameters as on the grating platform.

Initially, we hypothesized that adhesion might result from hydrophobic interactions between the methylated backbone of polystyrene and remaining methyl groups from the trimethylaluminum precursor used in the low-temperature ALD alumina process. However, thin polystyrene films spun directly onto alumina could be easily removed with tape (Figure 3a), indicating that there was no native adhesion between polystyrene and alumina. Hydrophobic interaction was further ruled out through testing with the hydrophobic silane TMCS (Figure 3b). Meanwhile, films coated onto phenyl-terminated P and N Silanes, as well as epoxy-terminated G silane, passed the tape test without issue (Figure 3c–e). The survival of all three films suggests two mechanisms of adhesion. First, phenyl-terminated silanes can interact through π–π interactions with the phenyl groups of polystyrene, forming multiple quadrupole interactions along the polymer’s length [29]. The extent of interaction in this arrangement depends on the organosilane density on the alumina surface and the orientation of the phenyl side group with respect to normal from the plane of interaction and reactive surface groups. Meanwhile, epoxy-terminated G silane lacks phenyl groups for such interaction, suggesting dipole–dipole interactions as the mechanism for that film’s survival since chemical reactions (i.e., grafting) between the epoxide side group and polystyrene have only been demonstrated at elevated temperature and for carboxy- or anhydride-terminated polystyrenes [28,30].

#### 2.1.3. Film Morphology over Ag Grating

The surface morphology of the thin polystyrene film determines the potential protein binding density in the bioassay. In the absence of an adhesion layer, the somewhat hydrophobic polymer may form nano-islands of polystyrene with minimal surface interaction. The fact that thicker (63 nm) polystyrene layers fully adhere to the P-, N-, and G-silanized alumina surfaces without forming islands indicates that the silanized surface properties are sufficient to prevent spinodal dewetting [31]. 

**Figure 4:**
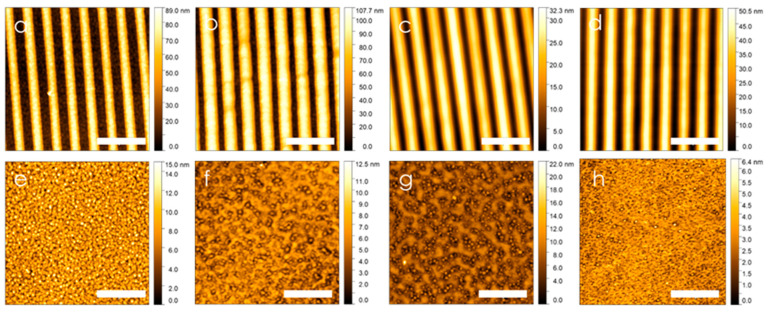
Atomic force microscopy of polystyrene over alumina-coated gratings (**a**–**d**) and flat silver (**e**–**h**): (**a**,**e**) as-prepared ALD alumina; (**b**,**f**) 5 nm polystyrene; (**c**,**g**) 7 nm polystyrene; and (**d**,**h**) 9 nm polystyrene. (Scale bar = 1 µm).

Atomic force microscopy (AFM) was used to analyze the surface morphology of thin polystyrene films on both the grating and flat alumina-coated silver surfaces (Figure 4) and average roughness (R_a_) summarized in Table 1. Polystyrene thickness was increased from 5 nm to 9 nm by adjusting the polystyrene concentration in solution, and we compared the resulting films to the as-prepared alumina-coated silver films. As-prepared alumina-terminated gratings (Figure 4a) exhibited the typical appearance of sputtered silver grains, with an average roughness R_a_ = 1.68 ± 0.02 nm for flat silver. We observed that polystyrene tends to fill the gaps between the grains, both on the flat and grating surfaces (Figure 4b,f). This filling effect reduced the surface roughness and covered the lower grains while leaving the taller grains exposed. Increasing polystyrene concentration improved the apparent coverage and accumulated in the groove areas, especially in the valleys of the grating structure. At 9 nm thickness, the film became significantly smoother (R_a_ = 0.52 ± 0.1 nm), with only the peaks of the grains remaining visible (Figure 4h). Notably, the maximum height difference on the flat silver surface reduced from approximately 15.0 nm for the as-prepared film to just 6.4 nm for the 9 nm film. This reduction in relative height of 8.6 nm closely aligned with ellipsometry data obtained for a similar film spun onto alumina-coated silicon. 

#### 2.1.4. Chemical Composition of the Films

**Figure 5:**
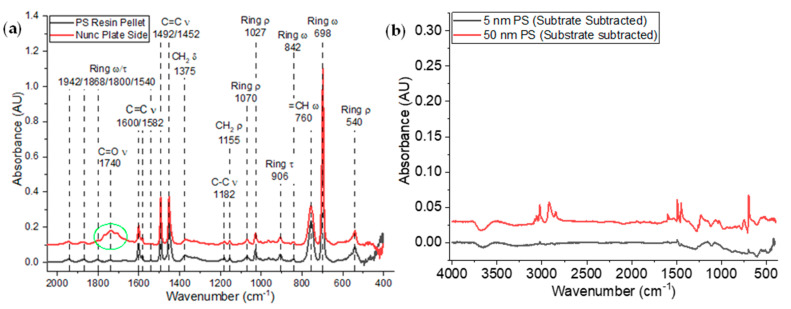
FTIR spectra of (**a**) commercial Nunc plates and PS resin and (**b**) 5 nm and 50 nm PS films.

We used FTIR to compare the chemical composition of the thin polystyrene films with that of the original polystyrene resin pellet and the surface of the commercially available Nunc Maxisorp^®^ polystyrene plates (Figure 5). One notable difference between the peaks observed in the commercial plate and the polystyrene resin is the presence of a large, broad peak at 1740 cm^−1^. This peak significantly overlaps with a smaller, broad resonance peak at 1800 cm^−1^ that is one of a quartet of peaks associated with ring bending at approximately 1942, 1868, 1800, and 1540 cm^−1^, respectively. The 1740 cm^−1^ peak corresponds to C=O stretching and signifies the oxidation of the carbon free radical generated through γ-irradiation of the Nunc Maxisorp^®^ surface. This surface treatment subsequently alters the surface energy of the Maxisorp^®^ surface, likely enhancing protein binding by attracting hydrophilic residues of folded and partially unfolded proteins. 

The as-prepared polystyrene thin films exhibit many of the prominent peaks observed in the polystyrene resin and the Nunc Maxisorp^®^ plate. These peaks become visible after the subtraction of the alumina and base silicon used in these experiments, specifically, the strong peaks ranging from 3250−2750 cm^−1^ associated with methyl stretching, a pair of doublets from 1600−1452 cm^−1^ associated with C=C bending, and the ring peak at 698 cm^−1^ are present in both the thicker 63 nm film and the 5 nm film. 

### 2.2. Comparative Fluorescence in Microplate Reader

**Figure 6:**
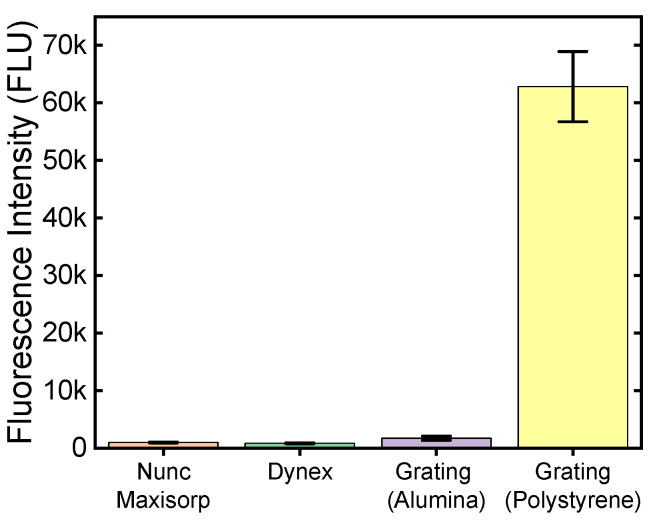
Fluorescence intensity of PS coated grating, grating without PS, and commercial plates.

Figure 6 presents the background-subtracted signal generated by binding of RRX-labeled goat anti-mouse antibodies compared across the four substrates using a fluorescence microplate reader: Nunc Maxisorp^®^ γ-irradiated polystyrene microwell plate, Dynex polystyrene microwell plate, our in-house fabricated alumina-terminated silver plasmonic grating, and polystyrene-terminated silver plasmonic grating. Microplate reader parameters including light source intensity, excitation and emission windows, and instrument gain were tuned such that same conditions could be used to extract meaningful fluorescence measurements across all four substrates without over- or under-saturating the detector. RRX-labeled proteins produced an average fluorescence of 984 FLU on the Nunc Maxisorp^®^ plate, 855 FLU on the Dynex plate, 1748 FLU on the alumina-terminated silver grating, and 62839 FLU on the polystyrene-terminated silver grating. 

Normalizing the fluorescence results using the Nunc Maxisorp^®^ plate as reference, it was observed that the Nunc Maxisorp^®^ plate exhibited a 13% higher fluorescence signal compared to the Dynex plate. Alternatively expressed in terms of “enhancement factor,” the Dynex plate yielded a value of 0.87× relative to the Nunc Maxisorp^®^ plate. This relative yield of fluorescence intensity between the Nunc Maxisorp^®^ and Dynex plates remained consistent across multiple experiments using separate plates and various reader parameters during the optimization process leading up to the current findings. In contrast, the alumina-terminated silver grating displayed a substantial enhancement factor of 1.78× when compared to the Nunc Maxisorp^®^ plate. Finally, the polystyrene-terminated silver grating exhibited an exceptional enhancement factor of 63.8× relative to the Nunc Maxisorp^®^ plate. 

**Figure 7:**
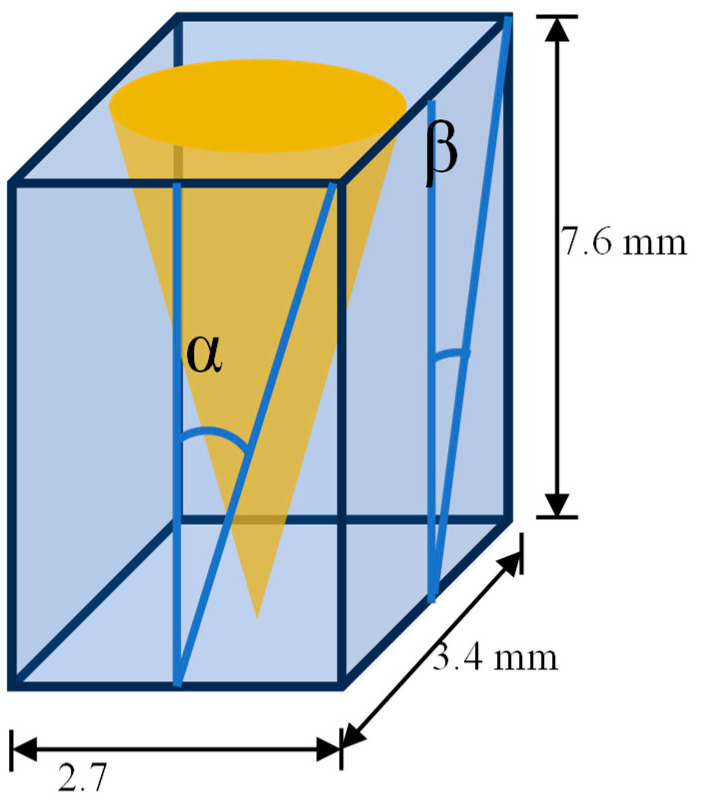
Schematic of the rectangular wells of the 24-well adapter showing the maximum incidence angles, α = 19.6° and β = 24.1°.

Competing physical properties contribute to the fluorescence enhancement factors observed above. Both Nunc Maxisorp^®^ and Dynex plates feature flat, unstructured polystyrene surfaces with high binding density and that minimize reflection and backscattering of excitation light, providing a dark background for visualizing isotropic fluorescence. Silver plasmonic gratings significantly enhance signal compared to same films on glass, but the alumina surface provides relatively sparse binding sites compared to polystyrene. Moreover, silver plasmonic gratings are highly reflective of s-polarized light at all angles as well as p-polarized light incident at angles far from the appropriate coupling angle for the wavelength of light in the given metal-dielectric plasmonic system, leading to an increased background signal compared to Nunc Maxisorp^®^ and Dynex plates. Fluorescence emission from the grating surface is anisotropic due to surface plasmon-coupled emission [32]. Using the dimensions of the microwell (Figure 7) and the dispersion relation [13], we can identify sets of appropriate excitation and emission wavelengths for the experimental conditions. Rhodamine Red X (RRX) was selected as the reporter dye because the coupling angles for excitation and emission bands of the dye were near normal to the grating plane in the grating-water system and, thus, guaranteed to be within the acceptance cone of the microplate reader. Subsequently, the emissive energy from the reporter dye is captured by the grating and re-emitted directionally toward the detector. The increased excitation due to plasmon coupling, high binding density of the polystyrene surface, and directional emission combine to produce the enhancement observed in these experiments. Considering the dispersion curve for polystyrene-terminated gratings in an aqueous buffer with a refractive index of 1.33–1.34, it is advisable to use Rhodamine Red X, Alexa Fluor 594, AlexaFluor 647, and similar dyes falling within the 500–700 nm excitation and emission range.

### 2.3. COVID-19 Single-Molecule Fluorescence Immunoassay

**Figure 8:**
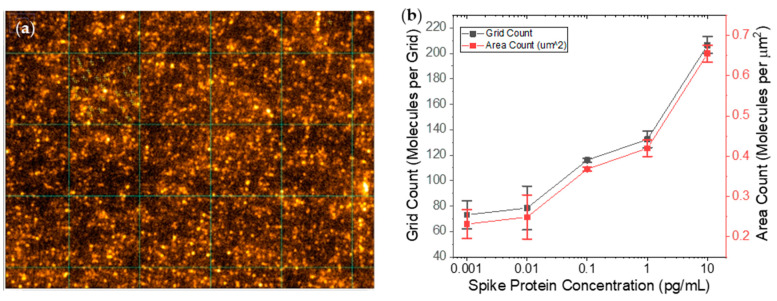
(**a**) A fluorescence image of 100 fg/mL of S-protein with 6 μm × 6 μm grids, and (**b**) single molecule counting from plasmonic grating with various concentrations of spike protein between 1 fg/mL and 10 pg/mL.

Single molecule blinking behavior was observed for all concentration of S-protein with an integration time of 2 s by using the CMOS camera. The number of S-protein molecules located within a 6 μm × 6 μm grid of the plasmonic sensor surface were counted by observation of single molecule blinking behavior over the course of a two-minute time trace (Figure 8a). Counts were averaged across 12 grids per well and 3 wells per concentration. Upon analyzing the S-protein movies that consisted of sixty frames, on plasmonic gratings at different concentrations, we observed the decrease of single molecules as the concentration of the spike protein reduced. The resulting emission intensities have been reported in Figure 8b. Based on Figure 8b, we find that it is possible to differentiate between S-protein concentrations from 10 fg/mL to 10 pg/mL.

## 3. Discussion

In this study, we have established a method of integrating polystyrene thin film coatings onto plasmonic silver gratings and demonstrated their use as a platform for ultra-sensitive detection of biomarkers for disease. The phenyl-terminated adhesion layer appropriately matches the surface energy of polystyrene and provides a noncovalent mechanism of adhesion that is shelf-stable and chemically robust to biomolecule solutions and surfactant buffers. The resulting polystyrene surface allows for biomolecule binding using the facile carbonate buffer method used in commercial assays, eliminating the need for downstream plasma processing required for the zero-length crosslinker chemistry used with alumina-terminated gratings. The binding of biomolecules on the polystyrene surface is more than 60× higher than γ-irradiated commercial microwell plates. Increasing molecular immobilization density improves the efficiency of any downstream capture, enzymatic, or other interactive activity with analytes of interest, ensuring a greater likelihood of visualizing target analytes or specific interactions from any given sample, regardless of its concentration. It further significantly expands the dynamic range, lowers detection limits, and improves mean accurate maximum concentration. 

A few challenges remain for further investigation into how processing of the thin polystyrene film impacts binding and enhancement. First, vacuum annealing at higher temperature reduces thickness of thin polystyrene film on silica. Whereas elevated temperature was avoided due to potential impact on silver, a balanced approach may benefit film stability and the reduced thickness decrease the distance between reporter dyes and the metal–dielectric interface. Second, irradiation to form carbon free radicals appears to improve binding to Nunc Maxisorp^®^ compared to unmodified plates. These radicals freely bind to oxygen in air, forming carbonyl species. Other forms of modification such as plasma processing can also oxidize the film, and may lead to improved binding compared to unmodified polystyrene-terminated gratings. Meanwhile, ultra-thin films possess an inherent tendency to restructure in response to internal stresses and evolving local environmental conditions. This restructuring process often changes the exposed surface groups. Plasma processing also etches surfaces, which could remove the entire ultra-thin film. Future study will determine the value of plasma processing for improving binding, as well as the stability of oxidative damage using this method. In sum, polystyrene-terminated silver plasmonic gratings hold promise as biosensor platforms for ultra-sensitive detection of disease biomarkers.

## 4. Materials and Methods

### 4.1. Grating Preparation

The gratings were prepared based on the previous work [9,16,18]. Briefly, a soft lithography process was used to fabricate alumina-terminated silver plasmonic gratings for further use. The grating master mold was prepared by curing polydimethylsiloxane (PDMS, 5:1 Sylgard^®^ 184, Fisher Scientific, Waltham, MA, USA) on a halved, cleaned HDDVD for 24 h at 50 °C and 55% relative humidity. Cured PDMS was cut into 1″ square slabs and used for stamping.

Just prior to stamping, plain glass microscope slides (Fisherbrand, Fisher Scientific) were pre-cleaned with dilute soap solution, rinsed in deionized (18.2 MΩ-cm) water, and dried under flowing nitrogen. Pre-cleaned slides were immersed in a 3:1 H_2_SO_4_:H_2_O_2_ (Piranha solution) for 15 min, washed twice in fresh deionized water, rinsed with copious deionized water, and finally dried under flowing nitrogen. Simultaneously, a 3% *w*/*w* polymer ink solution (GR650F, Techneglas, Perrysburg, OH, USA) in ethanol was spin-cast onto a PDMS slab and stamped onto the cleaned glass slides. The gratings then underwent vapor treatment with a 1:1 solution of 3-aminopropyltriethoxysilane in ethanol, followed by pre-baking at 60 °C for 3 h and baking at 400 °C for 1 h. Subsequently, a 100 nm layer of silver was deposited using an AJA RF Magnetron sputter system. Finally, a 10 nm alumina layer was deposited through low-temperature (65 °C) atomic layer deposition (ALD). Alumina-capped sliver gratings constitute the “as-prepared” alumina-terminated sample as further characterized below.

### 4.2. Polystyrene Thin Film Preparation

Trichloromethylsilane (TMCS), Phenyltriethoxysilane (P silane), *N*-Phenylaminopropyltrimethoxysilane (N silane), and 3-Glycidoxypropyltrimethoxysilane (G silane) were acquired from Gelest and employed without further alterations. As-prepared gratings and accompanying 10 nm ALD alumina on plain glass were exposed to 7 W CO_2_ plasma for 30 s to activate the surface, then immediately dip-coated in organosilane solutions with concentrations of 0.1%, 1%, 2%, or 5% *v*/*v* organosilane in toluene for 5 min. Silanized samples were washed in successive baths of fresh (1) toluene, (2) ethanol, and (3) ethanol for one minute each. After the final ethanol bath, the silanized samples were rinsed thoroughly with 2-propanol, dried under flowing nitrogen, and heated at 120 °C for 10 min. To prepare polystyrene films, Chevron MC3700 polystyrene pellets (Seaview Plastic Recycling, Bridgeport, CT, USA) were dispersed in toluene and sonicated to achieve a 10 mg/mL stock solution. The stock was diluted to 1 mg/mL, spin-coated onto the silanized gratings at 3000 rpm for 30 s, and subsequently vacuum annealed at room temperature for 1 h. Likewise, stock solution was spin-coated onto the silanized alumina on plain glass at 3000 rpm for 30 s, and subsequently vacuum annealed at room temperature for 1 h.

We employed various techniques to characterize the polystyrene films. An adhesion assessment was carried out using a tape test, which involved the firm application of Scotch^®^ tape to a section of the 50 nm polystyrene films on both silanized and unsilanized alumina on glass, followed by the swift removal of the tape in one even motion. Optical profilometry was utilized to capture images and determine the degree of film removal in each sample. Thickness and optical properties were measured using variable angle spectroscopic ellipsometry (VASE, J. A. Woollam). When feasible, we confirmed thickness and topography through atomic force microscopy (AFM) and optical profilometry. Finally, FTIR analysis was conducted to compare the chemical composition of the prepared polystyrene layer with commercial plates. 

### 4.3. Comparitive Bioassays

We analyzed and compared the bioconjugation of protein to our polystyrene-terminated gratings and as-prepared alumina-terminated gratings with two commercially available 96-well polystyrene microplates: Nunc Maxisorp^®^ and Dynex. Immediately prior to use, as-prepared alumina-terminated gratings were exposed to 7 W CO_2_ plasma for 30 s to activate the surface [18] while polystyrene-terminated surfaces were used without further modification. Subsequently, a 24-well slide adapter (Grace BioLab, Bend, OR, USA) was attached to each of the grating slides: plasma-treated as-prepared gratings or polystyrene-terminated gratings featuring either P, N, or G silane adhesion layer. 

For the plasma-processed as-prepared alumina-terminated grating, we created a solution containing 4 mg/mL EDC and 11 mg/mL Sulfo-NHS in 2-(*N*-morpholino)ethanesulfonic acid (MES) buffer at pH 6. Each well received 100 µL of this solution and was incubated at room temperature for 15 min. Next, we diluted antibody labeled with Rhodamine Red X fluorescent dye (Ab-RRX, Jackson ImmunoResearch, West Grove, PA, USA) to a concentration of 2 µg/mL in MES buffer at pH 8, adding 100 µL to each well, resulting in a final concentration and volume of 1 µg/mL and 200 µL, respectively [18]. For polystyrene-terminated substrates, we diluted Ab-RRX to 1 µg/mL in carbonate buffer at pH 8 and added 200 µL to each well, ensuring that the final volume and Ab-RRX concentration were consistent across all substrates. Subsequently, all samples were stored at 4 °C overnight, brought to room temperature for 30 min, and washed in triplicate using 1 M phosphate buffered saline with 0.1% Tween-20 surfactant (PBST) with gentle agitation on an orbital shaker. The final wash buffer was replaced with 200 µL of 1 M phosphate buffered saline for measurement. Measurements on all samples were taken using a BioTek H4 Synergy microplate reader in fluorescence mode. Excitation and emission were carried out using 555 ± 10 nm and 600 ± 10 nm monochromator bands, respectively. The detector height above the well and gain settings were optimized to ensure measurements were taken at the same gain level for all samples without saturating the detector or reaching zero signal. Specifically, the final adjusted detector height above the well was set at 6 mm for the Dynex and Nunc plates and 1 mm for the well-adapted gratings, with a consistent gain level of 135 for all samples.

### 4.4. COVID-19 Assay

The COVID-19 assay was designed as an indirect serological assay to detect anti-COVID-19 spike protein antibodies. In this assay, we fabricated 5-nm polystyrene-terminated gratings using N silane for the adhesion layer, following the procedures outlined in previous sections. Spike protein (S-protein) and monoclonal anti-COVID-19 S-protein antibody (CR3022) were purchased from Abcam (Cambridge, UK). Additionally, we obtained an RRX-labeled polyclonal antibody against CR3022 from Jackson ImmunoResearch Laboratories, Inc. S-protein was diluted to a concentration of 0.5 µg/mL in carbonate buffer at pH 8. Well-adapted polystyrene gratings were incubated with dilute S-protein overnight at 4 °C. Subsequently, they underwent a triple wash with PBST. Following this, the S-protein-coated gratings were treated with 5% bovine serum albumin (BSA) for 2 h at room temperature. 

Various concentrations (1 fg/mL, 10 fg/mL, 100 fg/mL, 1 pg/mL, 10 pg/mL, 10 ng/mL, and 10 µg/mL) of S-protein antibody were introduced into each well of the coated gratings and incubated for 2 h, followed by washing in triplicate with PBST. An RRX-labeled antibody at a concentration of 2 µg/mL was then applied to each well and incubated at 4 °C overnight, once again followed by a triple wash with PBST. The 24-well adaptors were removed, and thin cover slides were placed over the samples before measurement under an epifluorescence microscope (BX51WI, Olympus, Tokyo, Japan). The samples were imaged and at least 60 frames per sample recorded using an ORCAFlash 2.8 CMOS camera at 2 s integration time on the epifluorescence microscope using a 60× water-immersion objective. The acquired images were then analyzed using ImageJ to quantify the presence of S-protein molecules. 

## Figures and Tables

**Table 1:** Summary of average roughness corresponding to Figure 4.

Sample	Flat Silver (nm)	Grating Peak (nm)	Grating Valley (nm)
As-prepared	1.68 ± 0.02	2.61 ± 0.07	2.49 ± 0.04
5 nm PS	0.92 ± 0.12	3.98 ± 0.09	2.04 ± 0.08
7.5 nm PS	1.48 ± 0.09	1.12 ± 0.21	0.90 ± 0.03
9 nm PS	0.52 ± 0.1	1.61 ± 0.35	1.30 ± 0.14

## Data Availability

Data will be made available on request.

## References

[1] Balhara N., Devi M., Balda A., Phour M., Giri A. (2023). Urine; a new promising biological fluid to act as a non-invasive biomarker for different human diseases. URINE.

[2] Nair R.R., An J.M., Kim J., Kim D. (2023). Review: Recent progress in fluorescent molecular systems for the detection of disease-related biomarkers in biofluids. Co-Ord. Chem. Rev..

[3] Liu R., Ye X., Cui T. (2020). Recent Progress of Biomarker Detection Sensors. Research.

[4] Natarajan A., Beena P., Devnikar A.V., Mali S. (2020). A systemic review on tuberculosis. Indian J. Tuberc..

[5] Butler-Laporte G., Lawandi A., Schiller I., Yao M., Dendukuri N., McDonald E.G., Lee T.C. (2021). Comparison of Saliva and Nasopharyngeal Swab Nucleic Acid Amplification Testing for Detection of SARS-CoV-2: A Systematic Review and Meta-analysis. JAMA Intern. Med..

[6] Li M., Yin F., Song L., Mao X., Li F., Fan C., Zuo X., Xia Q. (2021). Nucleic Acid Tests for Clinical Translation. Chem. Rev..

[7] MacLean E., Kohli M., Weber Stefan F., Suresh A., Schumacher Samuel G., Denkinger Claudia M., Pai M. (2020). Advances in Molecular Diagnosis of Tuberculosis. J. Clin. Microbiol..

[8] Pandey P.S., Raghuwanshi S.K., Shadab A., Ansari M.T.I., Tiwari U.K., Kumar S. (2022). SPR Based Biosensing Chip for COVID-19 Diagnosis—A Review. IEEE Sens. J..

[9] Bhatnagar K., Pathak A., Menke D., Cornish P.V., Gangopadhyay K., Korampally V., Gangopadhyay S. (2012). Fluorescence enhancement from nano-gap embedded plasmonic gratings by a novel fabrication technique with HD-DVD. Nanotechnology.

[10] Chen B., Pathak A., Gangopadhyay K., Cornish P.V., Gangopadhyay S. (2015). Single-Molecule Detection in Nanogap-Embedded Plasmonic Gratings. Nanobiomedicine.

[11] Chen B., Wood A., Darr C.M., Bok S., Gangopadhyay K., McFarland J.A., Maschmann M.R., Gangopadhyay S. Single-molecule Imaging of Metallic Nanostructures on a Plasmonic Metal Grating Superlens. Proceedings of the 2018 IEEE International Conference on Bioinformatics and Biomedicine (BIBM).

[12] Darr C.M., Korampally V., Chen B., Gangopadhyay K., Gangopadhyay S. (2014). Plasmonic-enhanced conjugated polymer fluorescence chemosensor for trace nitroaromatic vapor. Sens. Actuators B Chem..

[13] Wood A., Mathai C.J., Gangopadhyay K., Grant S., Gangopadhyay S. (2017). Single-Molecule Surface Plasmon-Coupled Emission with Plasmonic Gratings. ACS Omega.

[14] Basuray S., Pathak A., Chen B., Menke D., Darr C.M., Gangopadhyay K., Cornish P.V., Gangopadhyay S. (2015). Single Molecule Oscillations of an RNA/DNA Duplex in a Plasmonic Nanocavity. J. Nanomed. Nanotechnol..

[15] Wood A., Bok S., Mathai J., Chen B., Suresh D., Gangopadhyay K., Grant S., Upendran A., Kannan R., Gangopadhyay S. Anti-Corrosive films on Silver Plasmonic Gratings for Fluorescence Imaging of Single Molecules and Cancer Cells. Proceedings of the Conference on Lasers and Electro-Optics.

[16] Darr C.M., Mathai C.J., Gangopadhyay K., Gangopadhyay S., Bok S. High Binding Density Coatings for Biomolecules on Plasmonic Gratings and Their Sensing Applications. Proceedings of the 2022 IEEE 22nd International Conference on Nanotechnology (NANO).

[17] Huang Y., Darr C.M., Gangopadhyay K., Gangopadhyay S., Bok S., Chakraborty S. (2022). Applications of machine learning tools for ultra-sensitive detection of lipoarabinomannan with plasmonic grating biosensors in clinical samples of tuberculosis. PLoS ONE.

[18] Wood A., Barizuddin S., Darr C.M., Mathai C.J., Ball A., Minch K., Somoskovi A., Hamasur B., Connelly J.T., Weigl B. (2019). Ultrasensitive detection of lipoarabinomannan with plasmonic grating biosensors in clinical samples of HIV negative patients with tuberculosis. PLoS ONE.

[19] Lerman M.J., Lembong J., Muramoto S., Gillen G., Fisher J.P. (2018). The Evolution of Polystyrene as a Cell Culture Material. Tissue Eng. Part B Rev..

[20] Kawamura Y. (2004). Effects of Gamma Irradiation on Polyethylene, Polypropylene, and Polystyrene. Irradiation of Food and Packaging.

[21] Curtis A., Forrester J., McInnes C., Lawrie F. (1983). Adhesion of cells to polystyrene surfaces. J. Cell Biol..

[22] Guruvenket S., Rao G.M., Komath M., Raichur A.M. (2004). Plasma surface modification of polystyrene and polyethylene. Appl. Surf. Sci..

[23] Wangkam T., Yodmongkol S., Disrattakit J., Sutapun B., Amarit R., Somboonkaew A., Srikhirin T. (2012). Adsorption of bovine serum albumin (BSA) on polystyrene (PS) and its acid copolymer. Curr. Appl. Phys..

[24] Chen Z., Li P., Zhang Z., Zhai X., Liang J., Chen Q., Li K., Lin G., Liu T., Wu Y. (2019). Ultrasensitive sensor using quantum dots-doped polystyrene nanospheres for clinical diagnostics of low-volume serum samples. Anal. Chem..

[25] Liu Y., Yu J. (2016). Oriented immobilization of proteins on solid supports for use in biosensors and biochips: A review. Microchim. Acta.

[26] Ashley K.M., Meredith J.C., Amis E., Raghavan D., Karim A. (2003). Combinatorial investigation of dewetting: Polystyrene thin films on gradient hydrophilic surfaces. Polymer.

[27] Choi S.-H., Zhang Newby B.-m. (2006). Suppress polystyrene thin film dewetting by modifying substrate surface with aminopropyltriethoxysilane. Surf. Sci..

[28] Luzinov I., Julthongpiput D., Malz H., Pionteck J., Tsukruk V.V. (2000). Polystyrene layers grafted to epoxy-modified silicon surfaces. Macromolecules.

[29] Rapold R.F., Suter U.W. (1994). Conformational characteristics of polystyrene. Macromol. Theory Simul..

[30] Jones R., Lehnert R., Schonherr H., Vancso J. (1999). Factors affecting the preparation of permanently end-grafted polystyrene layers. Polymer.

[31] Xie R., Karim A., Douglas J.F., Han C.C., Weiss R.A. (1998). Spinodal dewetting of thin polymer films. Phys. Rev. Lett..

[32] Lakowicz J.R., Ray K., Chowdhury M., Szmacinski H., Fu Y., Zhang J., Nowaczyk K. (2008). Plasmon-controlled fluorescence: A new paradigm in fluorescence spectroscopy. Analyst.

